# An Aide Memoire for a Balancing Act? Critiquing The ‘Balance Sheet’ Approach to Best Interests Decision-Making

**DOI:** 10.1093/medlaw/fwaa027

**Published:** 2020-10-22

**Authors:** Camillia Kong, John Coggon, Michael Dunn, Alex Ruck Keene

**Affiliations:** 1 Institute for Crime and Justice Policy Research, Birkbeck College, University of London 42 Store Street London WC1E 7DB; 2 Centre for Health, Law, and Society, University of Bristol Law School, 8-10 Berkeley Square, Bristol, BS8 1HH; 3 The Ethox Centre and Wellcome Centre for Ethics and Humanities, Nuffield Department of Population Health, University of Oxford, Big Data Institute, Li Ka Shing Centre for Health Information and Discovery, University of Oxford, Old Road Campus, Oxford, OX3 7LF; 4 39 Essex Chambers and Dickson Poon School of Law, King’s College London, 81 Chancery Lane, London, WC2A 1DD

**Keywords:** Balance sheet, Best interests, Judicial deliberation, Mental capacity law, Practical reasoning, Value incommensurability

## Abstract

The balance sheet is commonly used as a deliberative approach to decide best interests in Court of Protection cases in England and Wales, since Thorpe LJ in *Re A (Male Sterilisation)* described the balance sheet as a tool to enable judges and best interests decision-makers to quantify, compare, and calculate the different options at play. Recent judgments have critically reflected on the substance and practical function of the balance sheet approach, highlighting the practical stakes of its implicit conceptual assumptions and normative commitments. Using parallel debates in proportionality, we show that the balance sheet imports problematic assumptions of commensurability and aggregation, which can both overdetermine the outcome of best interests decisions and obfuscate the actual process of judicial deliberation. This means that the decision-making of judges and best interests assessors more generally could fail to properly reflect the *nature* of values at stake, as well as the *skills of practical judgment* needed to compare such values with sensitivity and nuance. The article argues that critical reflection of the balance sheet makes vital space for a more contextualised, substantive mode of deliberation which emphasises *skills of qualitative evaluation* towards enhancing *conditions of articulation* around the range of values involved in best interests decision-making.

## I. INTRODUCTION

Where a person is deemed to lack capacity to make a decision on her own behalf, the balance sheet is commonly used as a deliberative approach to decide what should be done, in her best interests, in Court of Protection cases in England and Wales.^1^ The balance sheet as a best interests decision-making tool in cases concerning health and welfare decisions for adults first emerged in *Re A (Male Sterilisation)*, where Thorpe LJ articulated a semi-quantitative exercise, looking at the potential gains or losses of a particular course of action. In his words,

[a]t the end of that exercise the judge should be better placed to strike a balance between the sum of the certain and possible gains against the sum of the certain and possible losses. Obviously only if the account is in relatively significant credit will the judge conclude that the application is likely to advance the best interests of the claimant.^2^

The suggestion, therefore, was that the balance sheet would enable judges to quantify, compare, and calculate the different options at play.

The influence of Thorpe LJ’s approach has been profound. He had anticipated that the procedure was only to be provisionally used ‘[p]ending the enactment of a checklist or other statutory direction’.^3^ Neither a checklist nor any other statutory direction has been forthcoming^4^ aside from the requirements of section 4 of the Mental Capacity Act 2005 (MCA)^5^ and many judicial decisions have subsequently upheld the balance sheet as the informal standard of best interests deliberation. More recent judgments, however, gesture towards growing critical reflection around the form, substance, and practical function of the balance sheet approach, with McFarlane LJ describing it as an ‘aide memoire … and not a substitution for the judgment itself’.^6^ Others, such as Hayden J, describe how ‘the balance exercise is qualitative rather than numerical’^7^ and, elsewhere, he states even more critically that the balance sheet approach is unhelpful because ‘[i]t does not really accommodate the enormity of the conflicting principles which are conceptually divergent’.^8^

Arguably, Hayden J’s observation about conceptual divergence has been operating as an implicit assumption within best interests decision-making: alongside the balance sheet approach is often a deliberative appeal to ‘magnetic factors’, whereby certain values or considerations are presumed to have priority or significant weight of some kind. The explicit challenge to some uses of the balance sheet in more recent judgments is notable, not only because it problematises the underlying consistency of these two deliberative tools working side-by-side in best interests decisions, but also in how it raises fundamental questions around the role of values and substantive practical judgment in deliberation about best interests under the MCA, as conducted by judges or legal, social work, and health and social care practitioners.

In this article, we demonstrate how recent disagreement around the form, substance, and practical function of the balance sheet has normative and practical significance. As we argue below, its normative significance is two-fold. First, there seems to be a growing recognition that best interests decision-making must depart from (even suggestions of) a simple calculation of benefits and losses, because by its nature, such deliberation appeals to goods and values that resist the questionable normative commitments implied in the concept of balancing, such as value commensurability, unitary notions of the good, or the existence of a single ‘cover value’. Secondly, statements by MacFarlane LJ, Hayden J, and others are an important first step in correcting a core conceptual misunderstanding underlying the way that the balance sheet has been applied thus far, which may help shed further light on limitations around the imagery and language of balancing more generally as a deliberative tool in best interests decision-making. The concept of balancing implies commensurability, in that it remains a possibility to quantify different options within a singular scale, based on a common measure or value. However, rendering values commensurable is different from *comparing* and *deciding* between conceptually divergent goods. As Hedley J aptly referred to his task in *Re GM*, ‘[t]his is very much a comparison of apples and pears and trying in the context of it to strike the best interests [sic] with as broad a view of those interests as it is possible to do’.^9^ Thus, the balance sheet as a more extreme iteration of the deliberative act of balancing is effectively being used as a tool of *comparability*, but whether it can discard the problematic assumptions of commensurability remains an important question that demands further scrutiny.

Critical comments by the judiciary might suggest movement away from the balance sheet in best interests deliberation in mental capacity jurisprudence. However, the requirements under section 5 of the MCA mean that judges in fact conduct only a very small proportion of best interests decision-making. When we look in more detail about how the statutory requirements have been fleshed out for those undertaking acts in connection to the care or treatment of P, it is important to note that the statutory Code of Practice to the MCA 2005—to which those discharging functions under the MCA must have regard—refers to the balance sheet at paragraph 5.62 (see also Section II below). Guidelines for legal, social, and health care practitioners also consistently recommend and endorse the balance sheet approach as a structured deliberative tool that is an essential part of the section 4 best interests checklist, with some explicitly referencing the approach of the judiciary, such as Thorpe LJ’s judgment in *Re A*.^10^ Its prevalent use in contexts prior to, as well as within, the courtroom setting thus suggests that there are important practical stakes in clarifying and resolving the conceptual and normative issues we have set out above. If the balance sheet is applied uncritically, best interests decision makers who use this device—including judges, barristers, solicitors, and social and health care practitioners—risk taking up implicit assumptions and commitments which could overdetermine the outcome of their best interests decisions, or which poorly reflect the actual process of deliberation. These commitments—and by default, the decision-making of best interests assessors—would then fail to properly reflect the *nature of* values and goods at stake, as well as the *appropriate mode of deliberation* or *skills of practical judgment* needed to compare these with sensitivity and nuance. Moreover, critical reflection on the various underlying commitments within the deliberative act of ‘balancing’ or ‘weighing’ as applied most rigidly within the balance sheet exercise may make vital space to explore the normative status and importance of other factors in best interests decision-making, such as the role and status of the values of P, the subject of proceedings. Our focus on the balance sheet in judicial best interests decision-making thus functions as an instructive gateway that helps contextualise and problematise its unreflective use in applications of section 4 of the MCA, in cases where its inclusion in submissions to judges help frame and inform their deliberations, as well as the many situations that do not even make it to the courts. It also serves as a stimulus for consideration of whether and how the balance sheet should be referred to in the next iteration of the MCA Code of Practice, which is under review at the time of writing.

The structure of the article is as follows. Section I provides a legal analysis of the balance sheet approach, as applied in the Court of Protection. Here we focus on providing a review of judicial disagreement around the function of the balance sheet, as well as the interplay between this deliberative approach, judicial values, and the values of P. In Section II the practical and conceptual problems with the balance sheet are critically analysed through reference to analyses of the practice and theory of proportionality, particularly in the application of human rights law. Rather than aim to advance new arguments around issues of balancing in debates about proportionality and value incommensurability in the law generally, we bring together these different perspectives to shed light on the deeper theoretical and conceptual warrant behind sceptical remarks within the judiciary that question the deliberative appropriateness of the balance sheet in mental capacity law. Section III accordingly delves deeper into the theoretical presuppositions of the balance sheet, drawing attention to the ways in which these presuppositions reveal a potential lack of fit between this approach and the nature of the goods at stake in best interests decision-making, as well as the necessity of practical reasoning and substantive evaluation in deciding between divergent values and goods. We then, in Section IV, apply our theoretical analysis to the question of whether judges and other best interests decision-makers can justifiably appeal to the balance sheet, and if not, to how they should reason about best interests in the process of their decision-making. To be clear, this article does not aim to critically analyse the best interests standard.^11^ Rather, our conclusions suggest that a critical lens towards the balance sheet will pave the way for a more contextualised, normatively expansive, mode of deliberation with particular emphasis on utilising evaluative language and advancing conditions of articulacy around the range of values involved in the deliberation of best interests.

## II. JUDICIAL DISAGREEMENT AROUND THE BALANCE SHEET

The legal genealogy of the balance sheet shows its transition from provisional advice, to its adoption as a standard deliberative tool in best interests decision-making in relation to children and incapacitated adults, and then to growing reservations amongst the judiciary. The balance sheet was introduced, as indicated above, by Thorpe LJ in *Re A (Male Sterilisation)*.^12^ The case concerned a young man with Down’s syndrome, A, who was being cared for by his mother. His mother was concerned that when, given her ill health, A moved into local authority care he might have a sexual relationship and be unable to understand the possible consequences. She applied for a declaration that a vasectomy was in his best interests. The judge at first instance found that while A was sexually aware and active, he did not understand the link between intercourse and pregnancy. However, he refused the declaration on the basis that the effect on A would be minimal. His mother appealed, which was ultimately unsuccessful, but Thorpe LJ took the opportunity to give some more general observations. He noted, relying upon the speeches of the House of Lords in *Re F*,^13^ that there could be ‘no doubt … that the evaluation of best interests is akin to a welfare appraisal [of a child]’.^14^ Thorpe LJ further reviewed the Law Commission’s report on mental (in)capacity law^15^ and the Government’s response^16^ to the effect that there had to be an ‘extensive’ evaluation of best interests.

In the passage then frequently cited thereafter, from which we have already drawn in the introduction, Thorpe LJ went on to say:^17^

[p]ending the enactment of a check list or other statutory direction [as to how best interests is to be evaluated] it seems to me that the first instance judge with the responsibility to make an evaluation of the best interests of a claimant lacking capacity should draw up a balance sheet. The first entry should be of any factor or factors of actual benefit. In the present case the instance would be the acquisition of foolproof contraception. Then on the other sheet the judge should write any counterbalancing dis-benefits to the applicant. An obvious instance in this case would be the apprehension, the risk and the discomfort inherent in the operation. Then the judge should enter on each sheet the potential gains and losses in each instance making some estimate of the extent of the possibility that the gain or loss might accrue. At the end of that exercise the judge should be better placed to strike a balance between the sum of the certain and possible gains against the sum of the certain and possible losses. Obviously only if the account is in relatively significant credit will the judge conclude that the application is likely to advance the best interests of the claimant.

Thorpe LJ noted that he suggested this approach:

only because [the first instance] judgment in the present case seems to me to concentrate too much on the evaluation of risks of happenings, some of which seem to me at best hypothetical. A risk is no more than a possibility of loss and should have no more emphasis in the exercise than the evaluation of the possibility of gain.

Although Thorpe LJ had expressly only indicated his approach pending the enactment of a check list or other statutory direction, when the MCA came into force on 1 October 2007, its attendant Code of Practice made express reference to *Re A* in a footnote to the underlined sentence in paragraph 5.62:

It is important that the best interests principle and the statutory checklist are ﬂexible. Without flexibility, it would be impossible to prioritise factors in different cases – and it would be difﬁcult to ensure that the outcome is the best possible for the person who lacks capacity to make the particular decision. Some cases will be straightforward. Others will require decision-makers to balance the pros and cons of all relevant factors. But this ﬂexibility could lead to problems in reaching a conclusion about a person’s best interests.

Whether because it was referred to in the Code of Practice or otherwise, the balance sheet has continued to be used in best interests decision-making for adults, in particular in medical treatment cases, even as interpretation of what it requires has inevitably evolved from Thorpe LJ’s original approach in line with increased case law decided under the MCA. A review of cases decided between 1 October 2007 (when the MCA 2005 came into force, and the ‘new’ Court of Protection started hearing cases) and 1 March 2019^18^ found that 64 cases made express reference to a balance sheet, and, in particular in medical treatment cases, observations often made along the lines that the use of such a tool is ‘conventional’.^19^ As had been done prior to the coming into force of the MCA 2005,^20^ some decisions have referred also to the balance sheet in the context of welfare decisions.^21^ In 2012, former Senior Judge Lush noted that:

When carrying out a best interests' analysis in health and welfare cases, judges of the Court of Protection generally apply what is known as “the balance sheet approach” and then look for any “factor of magnetic importance” which may tip the balance, so to speak.^22^

The reported cases do not, in fact, bear out the proposition that judges ‘generally’ apply the balance sheet approach, at least expressly, although Senior Judge Lush may well have been drawing upon his own personal, anecdotal, experience in a context where most decisions of the Court of Protection are not, in fact, encapsulated in a reported judgment.^23^ There are also reported cases in which the balance sheet has been used in cases concerning property and affairs, although in this context on occasion a closer examination of how the term is used shows that the balance sheet under consideration is, in fact, the balance sheet of factors that P would have taken into account.^24^

Despite its continued use, however, judges have expressed reservations as to the utility of the balance sheet approach. In some cases, this is because of the nature of the decision to be taken: most obviously in relation to decisions as to whether life-sustaining treatment should be continued in the context of a person in a permanent vegetative state, in which it has (questionably) been held that there is nothing to put in the balance in favour of continuing life.^25^ Former Senior Judge Lush also expressed his doubts as to the utility of the balance sheet in property and affairs cases concerning adults lacking the material decision-making capacity.^26^

More recently, the courts, in relation to both children and adults, have cautioned against the uncritical or simplistic reliance on the balance sheet. In *Re F (Children)*, McFarlane LJ (now President of the Family Division and Court of Protection) stated that he:

entirely agree[d] that some form of balance sheet may be of assistance to judges, its use should be no more than an aide memoire of the key factors and how they match up against each other. If a balance sheet is used it should be a route to judgment and not a substitution for the judgment itself. A key step in any welfare evaluation is the attribution of weight, or lack of it, to each of the relevant considerations; one danger that may arise from setting out all the relevant factors in tabular format, is that the attribution of weight may be lost, with all elements of the table having equal value as in a map without contours.^27^

Perhaps unsurprisingly, as judges of the High Court sitting as Court of Protection are very largely drawn from those with a background in cases concerning children, such judges have expressed similar concerns. In *Cambridge University Hospitals NHS Foundation Trust v BF*,^28^ Macdonald J noted that the approach in *Re A* was advanced ‘pending the enactment of a checklist or other statutory direction’. Citing *Re F*, he continued: ‘Within this context, whilst the balance sheet is a very useful tool, having compiled the same the court must still come to its decision as to best interests by reference to the principles set out above grounded in the Mental Capacity Act 2005 s. 4.’ The former Vice-President of the Court of Protection, Charles J, also noted a similar reservation,^29^ and the current Vice-President, Hayden J, has been more trenchant. In *NHS Foundation Trust v QZ*, a serious medical treatment case, he observed that the case before him was not one where the *Re A* balance sheet was helpful. In a *dictum* quoted in the introduction to this judgment, he held that the balance sheet ‘does not really accommodate the enormity of the conflicting principles which are conceptually divergent’.^30^ He continued:

It is very clearly established that the approach to evaluating the best interests of P in these circumstances, under the framework of the Mental Capacity Act 2005, are the principles set out in section 4 of that Act. It is also, as Ms Roper emphasised in her position statement, well established that there is no constraint on the factors the court should take in to account when considering best interests. She emphasises this is an holistic exercise involving not only medical best interests but the wider social and emotional gamut of a patient's interests. Although Miss Watson invites me to take a ‘balance sheet’ approach to the exercise, such a process is, in my judgement, as MacFarlane LJ has stated [in *Re F*], rather like a reading a map without contours, different factors plainly weigh disproportionately.^31^

## III. HUMAN RIGHTS, JUDICIAL COMPETENCE, AND JUDGMENTS OF PROPORTIONALITY

The foregoing genealogy of the balance sheet in the case law reveals diverging views amongst the judiciary: though initially conceived as a list to help draw out ‘pros’ and ‘cons’, the balance sheet has also evolved into a tool that suggests the weighing up of competing options. On one hand, it remains a ‘conventional’ tool that is frequently endorsed as an appropriate deliberative mechanism to make best interests decisions, not least within the current version of the MCA Code of Practice and in numerous practice guidelines for social and health care practitioners.^32^ On the other hand, dissenting (and influential) voices amongst the judiciary, including the current President and Vice-President of the Court of Protection, question its utility and function. The more assertive statements cautioning the application of the balance sheet tool highlight two notable features: first, certain judges are explicitly arguing for the application of practical judgment in judicial deliberation in ways that extend beyond the mechanistic act of balancing factors or weighing up of options. The fact that these voices caution against adopting a mechanistic approach expresses the worry that this is indeed how the balance sheet in best interests decision-making is being deployed in practice—whether amongst different judges, or the legal, healthcare and social care practitioners charged with carrying out a balance sheet exercise as part of the best interests checklist in their submissions. The rigid template in numerous practice guidelines of the balance sheet^33^ attests to the temptation to treat the exercise mechanistically, which may potentially explain the perceived need of certain members of the judiciary to explicitly reject such an approach in recent judgments. Secondly, there is a recognition that the nature of the cases and goods at stake in best interests decision-making is poorly captured through the imagery and act of balancing and weighing—thus raising critical questions about why judges remain committed to this language and indeed, the balance sheet, despite recognising the temptation towards, and inherent limitations of, overly literal depictions of balancing in deliberative tools.

These two issues echo longstanding parallel debates regarding the meaning of balancing in the proportionality doctrine, particularly in the context of constitutional and human rights adjudication, further grounding the practical—and indeed principled—concerns expressed amongst the judiciary.^34^ Such debates may also help explain the ambivalence and contradiction in judicial critiques of the balance sheet—rejecting its mechanistic overtones, on one hand, while cleaving to it as a deliberative tool, on the other. We thus frame discussion of the practice and theory of proportionality in this section to contextualise and bring to the forefront similar presuppositions that are embedded within the balance sheet in mental capacity law. Following others, such as Paul Craig, who challenge the exceptionalisation of debates concerning judicial values and institutional competence in public law contexts,^35^ we are similarly interested in exploring how existing criticisms of balancing in the proportionality doctrine may be brought to bear on decision-making tools within mental capacity law—specifically drawing out the deeper grounds on which we might question the use of the balance sheet as a deliberative tool for best interests decision-making on behalf of individuals who are deemed to lack capacity.

The relevance of proportionality adjudication may not be immediately apparent in the context of mental capacity law. However, the European Convention on Human Rights forms the backdrop to adjudication under the MCA. This is so in particular because the Court of Protection is itself a public authority under the Human Rights Act 1998, and thus bound not to act incompatibly with P’s Convention rights.^36^ The Court is, furthermore, bound to follow principles of statutory interpretation to ensure, insofar as possible, that the law is applied compatibly with P’s Convention rights.^37^ And the relevance of principles drawn from human rights adjudication is practically unavoidable because so much mental capacity jurisprudence operates within the ambits of key Convention rights, such as Article 3 (the absolute right not to be subject to inhuman or degrading treatment), Article 5 (the limited right to liberty and security if the person), and, of particular interest for the purposes of the current discussion, Article 8 (the qualified right to respect for private and family life). As Sir Mark Hedley observes in relation to the application of Article 8, proportionality provides a background rule that dictates that ‘the least intervention [is] consistent with best interests’ and may even demand a wholesale reassessment of those best interests.^38^

As well as providing relevant direct points of context, we might also consider how judicial evaluation of Article 8 impacts on non-MCA cases wherein judges are nevertheless required to undertake balancing exercises to determine questions associated with the identification, weighting, and weighing of substantive personal values, individual autonomy, and concerns for a person’s welfare. Questions spanning adjudication in this context include, for example, questions of whether there is a right positively to define one’s own best interests in a medical context;^39^ challenges concerning rights associated with ‘assisted dying’;^40^ determinations of whether there is a right to smoke tobacco;^41^ or a right to have access to a needle exchange programme for recreational drug use.^42^

There is a wealth of literature that explores judicial approaches to proportionality assessments. With the advent of the Human Rights Act, the courts may be seen to have been afforded a role as a check on executive activity that is more intensive than was the case given the previously existing grounds of judicial review. This raises questions of both constitutional and institutional competence, each underpinned by critiques of the nature of decision-making that is appropriately undertaken by the courts.^43^ Lord Steyn’s *dicta* in the case of *R (Daly) v Secretary of State for the Home Department*^44^ explain the position, and how and why ‘the intensity of review is somewhat greater under the proportionality approach’ than under the previously existing grounds of judicial review:^45^

The contours of the principle of proportionality are familiar. In *de Freitas v Permanent Secretary of Ministry of Agriculture, Fisheries, Lands and Housing* [1999] 1 AC 69 the Privy Council adopted a three stage test. Lord Clyde observed, at p 80, that in determining whether a limitation (by an act, rule or decision) is arbitrary or excessive the court should ask itself:

‘whether: (i) the legislative objective is sufficiently important to justify limiting a fundamental right; (ii) the measures designed to meet the legislative objective are rationally connected to it; and (iii) the means used to impair the right or freedom are no more than is necessary to accomplish the objective.’

Clearly, these criteria are more precise and more sophisticated than the traditional grounds of review. What is the difference for the disposal of concrete cases? … I would mention three concrete differences without suggesting that my statement is exhaustive. First, the doctrine of proportionality may require the reviewing court to assess the balance which the decision maker has struck, not merely whether it is within the range of rational or reasonable decisions. Secondly, the proportionality test may go further than the traditional grounds of review inasmuch as it may require attention to be directed to the relative weight accorded to interests and considerations. Thirdly, even the heightened scrutiny test developed in R v Ministry of Defence, Ex p Smith [1996] QB 517, 554 is not necessarily appropriate to the protection of human rights.^46^

The focus in human rights claims raises substantive questions about whether there has been an interference with an applicant’s Convention right(s), rather than attention to the process by which a decision was reached. And if the proportionality of a decision is under issue, the question, following the test as stated in Lord Steyn’s speech above, requires a balancing exercise. As Lord Bingham held in *R (Begum) v Denbigh High School Governors*,^47^ having given direct reference to Lord Steyn’s words:

There is no shift to a merits review, but the intensity of review is greater than was previously appropriate, and greater even than the heightened scrutiny test …. The domestic court must now make a value judgment, an evaluation, by reference to the circumstances prevailing at the relevant time[.]^48^

Importantly, the extent to which balancing represents a mechanistic or impressionistic exercise is unresolved in these judicial statements and likewise remains subject to vigorous debate in jurisprudential theory. Formal proponents, such as Robert Alexy, depict balancing as a legally constrained decision-making procedure that helps judges to arithmetically determine the conditional relation of precedence between competing principles within the particular circumstances of a case.^49^ While rejecting balancing within the precision of a scale of infinitely expressible gradations between 0 and 1, Alexy advances a ‘Weight Formula’ that allows judges to express legal propositions against weightings of (say) light, moderate, and serious. Thus, rights are permitted to be weighed through a method that permits of numerical representation and to be balanced with ‘concrete weight’ given to them within the factual context of the particular case. In this way, the weighing of interests is treated as a quantifiable, comparative procedure,^50^ purportedly arbitrating competing interests and principles using the language of ‘more’, ‘less’, ‘equal’, without appeal to substantive moral reasoning or non-legal, subjective values.^51^ Indeed, ‘it does not locate principles in a distant and unreachable heaven of values, but drafts them down from their abstract, and one might say, principled level, in order to render them fruitful by way of a process of rational argumentation in the individual case’.^52^

This mechanistic model is not without its critics, however, with both defenders and opponents of proportionality quick to ‘rescue’ an impressionistic model of balancing, or abandon balancing altogether as a mode of judicial decision-making. Common amongst these critics, however, is a shared scepticism that balancing can achieve the level of mathematical precision purported in formal accounts and that such precision is even desirable where conflicting rights, interests, and principles are at stake. As Kahn writes:

The concept of ‘balancing’ is itself both a metaphor and an abstraction. The metaphor is ambiguous. It describes both a process of measuring competing interests to determine which is ‘weightier’ and a particular substantive outcome characterized as a ‘balance’ of competing interests. The abstract concept of balancing, furthermore, tells us nothing about which interests, rights, or principles are weighted or how the weights are assigned.^53^

Critics of the formal approach to proportionality charge that this mechanistic approach to balancing incorrectly presumes a common metric and commensurability of interests and rights which misrepresents the nature of the goods at stake.^54^ Even without the literal arithmetic formulas espoused in formal approaches to balancing, the image of the scales is deeply evocative of a single cover value capable of weighing up opposing rights and interests. Not only does this reflect a fundamental category mistake—attaching the quality of sameness to that which is qualitatively different—but the consequences are equally worrying, whereby quantification implies the deliberative logic of trade-offs and optimisation requirements, resulting in human rights being ‘balanced away’ or ‘sacrificed’.^55^ This deliberative logic as such is fundamentally inappropriate to the rights, principles, and interests at issue.

Moreover, if no single cover value can measure divergent rights, interests, or goods, then deciding between them will involve a complex and intuitive deliberative process that goes beyond a legally constrained, formal process removed from substantive content. The formal understanding of balancing mistakenly suggests that the deliberative process is a quantitative rather than evaluative exercise, yet the complex nature of competing rights and values necessitates practical judgment that is flexible, open-ended, and capable of being responsive to changing circumstances. Balancing thus falls short of the promise of providing genuine insight into what happens in the deliberative ‘black box’. Though its formal proponents imply deliberation is reducible to an algorithm which purportedly generates legally constrained, justifiable results, in reality, what emerges instead is ‘a characteristically impressionistic assessment of the relative weights of competing considerations, which does not lend itself to rational reconstruction of the argumentative path that has led to a particular decision’.^56^ In other words, the misleading imagery and procedures of balancing hide the actual substance of judicial deliberation which involves the qualitative evaluation of conflicting principles or values.^57^

As already indicated above, judges at the highest level have stated within the body of their *dicta* how values inevitably are at play, with judgments of proportionality reached through the qualitative evaluation of these values. The nature of such qualitative evaluation is provided in Section III of this article, but a striking representation, in relation to the balancing of precisely the sorts of values that might feature in a Court of Protection best interests assessment, can be brought if we contrast the reasoning of the majority and minority in the Court of Appeal in *R (N)* v *Secretary of State for Health*.^58^ Here, the court was considering whether detained psychiatric patients should, under Article 8, enjoy a right to smoke: a right, if it existed, that they could not enjoy both by virtue of the policy of the NHS Trust under whose care they were detained, and by virtue of the expiration of an exemption that had been provided through Regulation 10 of the Smoke-free (Exemption and Vehicles) Regulations 2007.

Establishing whether such a right existed was inevitably going to be an evaluative exercise, with the majority ultimately holding that: ‘Difficult as it is to judge the importance of smoking to the integrity of a person’s identity, it is not, in our view sufficiently close to qualify as an activity meriting the protection of article 8.’^59^ However, the majority continued, *obiter*, to consider and present reasons why, had there been an interference, it would have been proportionate:

[T]here is strong evidence of the dangers of smoking both to smokers and to those subject to SHS [second-hand smoke] and powerful evidence that in the interests of public health a complete ban was justified in appropriate circumstances. We further agree that substantial health benefits arose from the ban and, as experience has shown, that the disbenefits were insubstantial. As to SHS, there has emerged powerful evidence of its dangers which supports the trust’s case on justification in a way which might not have been the case in the past. In all these circumstances we agree that the trust's policy would be justified under article 8(2) if article 8 were engaged at all.^60^

That a judge might come to an alternative view is unarguable, given the dissenting opinion of Keene LJ, who held that the regulations that permitted a ban for detained patients in secure psychiatric hospitals did indeed breach Article 8:

To a non-smoker like myself the importance of the activity of smoking to someone who smokes regularly or who may have smoked for many years is not easy to gauge, but it is apparent that to such people it is a pastime greatly valued. One knows of writers and journalists who cannot write without smoking, while others seem to obtain great personal pleasure from smoking after eating or other activities. A large percentage of the adult population seems unable to work without smoking at intervals during the day. Less anecdotally, there is the implicit assumption by the Joint Committee on Human Rights in its Sixth Report that smoking is an activity, interference with which would engage Article 8 …. It seems to me that for many people it forms an important part of their personal lives and possesses a value which reaches a level which qualifies for protection under Article 8 as part of their personal autonomy. …^61^

Keene LJ’s assessment of proportionality similarly highlights the complexities of such a balancing exercise:

I readily acknowledge that, in assessing proportionality in a matter like this, weight has to be attached to the position endorsed by the democratically-elected body. However, nothing put before this court demonstrates that Parliament ever appreciated that in reality the consequence of Regulation 10(3), the time-limit on exemption for mental health units, was likely to be a complete or virtually complete ban on smoking for those detained in secure mental hospitals. There was no debate on the merits of such an outcome, which means that there has been no democratic endorsement of it. Ultimately, the decision is one for the court. In the light of the matters to which I have referred, it seems to me that the prohibition in England on smoking in institutions like Rampton, a prohibition which results from the cessation of the exemption in Regulation 10 plus the security considerations applicable there, is more than is necessary to accomplish the public health objective of protecting people against second-hand smoke. It is therefore disproportionate, and there is a breach of Article 8.^62^

The approach to legal reasoning in this case (one of many from which we may have drawn) demonstrates the strengths, in practice, found in the critics of formal approaches to proportionality. Some defenders of proportionality, however, incorporate those criticisms to expound on a more informal, intuitive approach to balancing, arguing that the more formal model departs from the reality of its judicial application. For instance, Waldron describes the coherence between claims of weak incommensurability and the procedure of balancing, where the ordinary meaning of weighing up divergent goods implies ‘the reasoned articulation of our moral principles and priorities’ rather than quantification about competing values.^63^ Balancing as a judicial tool can therefore be understood in this ordinary sense rather than the formal approach to proportionality.^64^ Möller likewise pushes against the assumption that proportionality imposes a common metric on rights and principles. Instead, balancing functions as shorthand for the open-ended, unconstrained moral deliberation that reasons between principles, values, and interests characterised by weak incommensurability.^65^

But in the process of incorporating the incommensurability objection against proportionality, balancing becomes a catch-all deliberative tool, effectively trivialising its distinctive legal function. As Urbina writes, ‘[p]roportionality cannot simultaneously allow for open-ended moral reasoning about the right decision to the case – thus avoiding the objections made to the maximization version of proportionality and provide legal guidance – thus obtaining the benefits of legally directed adjudication’.^66^ To put a slightly different slant to Urbina’s point, moves to ‘save’ balancing from its critics risk distortions from two directions. From one direction, balancing loses its specialised legal function and continues to mask the actual phenomenon of moral judgment which carries the actual deliberative burden in evaluating divergent principles, rights, and interests. From another direction, we risk distorting the distinctive nature of the rights, principles, etc. at stake, because the evocative imagery of balancing continues to oversimplify the complexity of these goods.

Returning to mental capacity law, the controversies and debates around the theory and practice of balancing in the proportionality doctrine can be said to track the broad contours of judicial discomfort around the use of the balance sheet in best interests decision-making. For example, similar concerns of value incommensurability are expressed in Hedley J’s reference to comparing ‘apples and pears’ and in Hayden J’s identification of conceptually divergent, conflicting principles. Likewise, the pervasive force of optimisation and quantification connotations in balancing are evident in judicial statements questioning the function of the balance sheet exercise. Holman J, for example states in *A and B v Rotherham Metropolitan Borough Council*:^67^

I have read and re-read those ‘balance sheets’ and all the written closing submissions, and I have all the points listed there in mind. Judges frequently use the language of ‘balance’ and ‘balance sheets’ (and I do myself. I think lists such as the above are indeed very helpful). But the analogy with balancing scales may be misleading. When weights or objects are put on either side of a scale, their individual precise weights are known, or ascertainable. You can put four objects in one scale pan and seven in the other, and the scales will come down one way or the other due to the aggregate of the individual precise and ascertainable weights on each side. In a case such as this, however, none of the factors have precise weights. All that may be said of any individual factor is that, as a matter of judgment, it is more or less important or weighty than another.^68^

Holman J’s statement recognises the impressionistic as opposed to mechanistic nature of deliberation around the complex matter of best interests. It might be the case that judges do indeed refer to the balance sheet as a shorthand for an ‘aide memoire’, or the type of commonsense, intuitive comparisons we do on a regular basis in making practical judgments, seemingly akin to the weak incommensurability argued for by Waldron. Nonetheless, the criticisms expressed in the judgments of Hayden J, MacFarlane LJ, and Hedley J reveal the difficulty in shedding the figurative connotations of mechanistic deliberation, quantification, and commensurability in the judicial practice of balancing, even as Waldron and Möller eschew its reductive assumptions. For this reason, we share Urbina’s scepticism that balancing more generally remains an apt legal tool that makes sufficiently explicit the impressionistic determinations of judges.^69^

Equally, conceding to the impressionistic nature of judicial decision-making raises the additional spectre of longstanding critiques that best interests judgments simply amount to judges and other decision-makers expressing subjective opinions that are imposed on others. For example, Ian Kennedy strongly criticises the best interests test as ‘not really a test at all’ but a ‘crude conclusion of social policy’ that ‘allows lawyers and the courts to persuade themselves and others that theirs is a principled approach to the law’ which amounts to a form of ‘ad hocery’.^70^ Through the veneer of a contextualised approach which effectively atomises the law, he continues:

The court can then respond intuitively to each case while seeking to legitimate its conclusion by asserting that it is derived from the general principle contained in the best interests formula. In fact, of course, there is no general principle other than the empty rhetoric of best interests; or rather, there is some principle, but the court is not telling. Obviously the court must be following some principles, otherwise a toss of a coin could decide cases. But these principles, which serve as pointers to what amounts to best interests, are not articulated by the court. Only the conclusion is set out. The opportunity for reasoned analysis and scrutiny is lost.^71^

Though Kennedy’s criticisms emerge in a context where best interests was either interpreted under the ‘substituted judgment’, pre-MCA Chancery regime, or risk-driven analysis under the Mental Health Act, they nonetheless articulate clearly the potential dilemma facing judges and best interests decision-makers more generally once formal deliberative mechanisms, like the balance sheet, are explicitly rejected. And indeed, we would agree with Kennedy’s analysis to some extent: deliberative principles, such as balancing and its expression through the balance sheet, do indeed obscure the deeper reasons and values motivating a best interests analysis. However, we would suggest that this indicates a promising direction, where best interests deliberation becomes geared towards the more transparent articulation of values underlying such complex decisions, rather than revealing a domain of ad hoc, subjective judgments or the innate emptiness of the best interests standard. But to properly harness this potential, it is particularly urgent to clarify the reasoning behind these more complex judgments in mental capacity jurisprudence: how do values and valuing impact these judgments? How does this practical reasoning look—what does it means to judge certain factors as possessing ‘magnetic significance’, compared to other factors? What are the phenomenological and normative features of such evaluative deliberation? We explore these aspects further in the next section.

## IV. VALUES AND PRACTICAL REASONING

Thus far, questions and contradictory views around the theory, function, and substance of balancing in proportionality help hone in on analogous issues within adopting the balance sheet approach in mental capacity law. One might argue that this means ‘saving’ balancing from more reductive formulations to capture more commonsensical intuitions about comparing and deciding between competing goods—as attempted by Waldron and Möller. Judicial unease expressed about the mechanistic assumptions implicit in the balance sheet indicate, however, that the problem with balancing goes beyond a simple matter of linguistic or definitional retrieval—that the formal, quantitative connotations evoked in its imagery, and enshrined in *dicta* such as Butler-Sloss P’s assertion in the case of *Re S (Adult Patient: Sterilisation)* that ‘the best interests test ought, logically, to give only one answer’,^72^ are difficult to dislodge in practice.

Ultimately, the deeper problem with the balancing imagery lies in the fact that it obscures and limits the scope of legitimate reasons which contribute to the decision-making process. Formal approaches to balancing prove to be so attractive because they attempt to provide a clear formula to decide between difficult options in accordance with a process that (at least) aspires to be as value-neutral as possible, where adherence to rule-of-law constraints and the avoidance of extra-legal values in judicial reasoning are upheld as laudable legal virtues. From this standpoint, the domain of moral values is dismissed as expressing ‘subjective’ or extraneous considerations that are irrelevant to hard legal obligations. However, the normative force of competing rights and goods commonly adjudicated in the legal sphere is derived from moral values that not only resonate within the socio-cultural context, but often involves valuing them as irreducible goods which resists any benefits-loss calculus or trade-offs. Though welfare decisions revolve around determining what is in the best interests of a single individual, divergent rights and goods often get at the very heart of what is often thought to be valuable in human life, such as self-determination, physical and emotional well-being, human intimacy and contact. It may well be true that some of these values have become so incorporated in the law that it becomes difficult to demarcate their source.^73^ Even in those cases, disengaging from various sources of value—whether they be moral, socio-cultural, religious, and so on—does not guarantee better, more justifiable reasoning, or a just outcome for individuals. What results instead is the distortion of what motivates our principles, rights, interests, and human goods, and why these are held in high regard. As these values become removed from their animating sources, the ensuing judgment appears as an unmoored legal declaration, impoverished of crucial expressive and justificatory resources.^74^

For example, Sir Mark Hedley writes eloquently about the deliberative nuances of making difficult decisions in welfare jurisdictions, such as the necessary task of identifying relevant and sometimes conflicting values in welfare cases. The fact that these values are distilled through the interpretive prism of rights does not remove the reality of the underlying values at stake, nor that judges need to decide between them. As he suggests, ‘these areas are deeply affected by human emotions, which may not be effectively constrained by rationality but could be seriously constrained by what is practicable’.^75^ Rather than shy away from the value-laden nature of best interests decisions, he argues that it is the judge’s role to address competing sets of values, represented by those of society, the person who is subject of proceedings, the family or families, and the judge herself.^76^ Often identifying these values will be elusive and shifting, but it is notable that Sir Mark Hedley states,

[W]e cannot ignore the values of the judge. It is unlikely that they will be referred to in the judgment; indeed, they may just be those cultural assumptions of which we are so unaware or at least do not articulate. My background has presumably imbued me with assumptions that have simply become part of me and, however self-aware I try to be, I will not spot everything. That will not stop them affecting my thinking. All this must go into the welfare pot.^77^

This means that ‘best interests are not purely rational’, demanding ‘the exercise of discretion [that] involves a deeply human judgment that may not always accord with purely rational assessment [but is] governed neither by culpability nor sentiment’.^78^

Sir Mark Hedley moreover calls into question the illusion of some objective weighting to values: actual practice reveals that taking seriously the conflicting values or goods in best interests decisions involves rejecting this kind of putatively precise measurement. Consider the common dilemma of determining the best interests of whether an older person with dementia should stay at home or be placed into care against their wishes. When moral values are excluded, it is easy for the logic of risk aversion and optimisation to take hold—the predominant reasons which tend to motivate public bodies. He writes:

Such a culture is always likely to prioritise physical protection because that is what can be measured. In a care home, the protected person will be kept warm, clothed, fed and provided with stimulating activity. That can be seen and measured, but emotional deprivation in sadness, isolation, and the loss of sense of belonging are not readily susceptible to measurement. Likewise, at home squalor can be measured (and professionals criticised) but a sense of security cannot.^79^

The lure of measurement lies in its impression that disparate goods can be maximised, reaching a concrete, ‘right’ answer. The practical reality, however, is that in deliberating difficult cases involving conflicting rights, where there is ‘more than one reasonable answer but only one decision’^80^ a judge is left with a core question: ‘Was I right? I simply do not know … [T]he breadth of the human condition affected by those rights means that their actual outworking (rather than their basis) is somewhat diffuse’.^81^

Sir Mark Hedley’s observations attest to the difficulty of identifying (or even *being able* to identify) the value sources that inform their judgment. But they also gesture towards two other interconnected points about evaluative attitudes and practical reasoning respectively. First, the elusiveness of measurement tells us something about the very nature of, and judicial evaluative attitudes towards, the goods at stake in difficult best interests cases. Limiting or excluding different sources of value from the space of legitimate judicial reasons would effectively express a failure to value certain rights, goods, and principles in ways that are fitting to the profound issues at stake in best interests cases. In the scenario of the older gentleman, for example, it means allowing the logic of risk management—and thereby measurable outcomes—to have *a priori* significance, disregarding goods which resist measurement, such as the equally valid and important claim of emotional security and belonging. In other words, drawing from a richer, more substantive pool of reasons indicates that one is attuned to the ways in which particular circumstances require valuing things *in the right way*—as appropriate to the kinds of goods that they are, ie as situated within a socio-cultural context, imbued with personal and intersubjective significance and potential moral connotations.^82^

Closer examination of the evaluative attitudes adopted in claims of commensurability and incommensurability help flesh out this point. Commensurability implies the possibility of extrinsic valuing, in so far as goods are not good in and of themselves but help maximise or achieve something else. Their value is therefore derived from an external source. As Anderson explains, extrinsic values fall under an *aggregative* principle which prescribes the maximisation of a particular value; the total amount of value dictates the appropriate response. Values as such are scalar and used to assign weights to certain goods, in order to rationally decide between which is ‘weightiest’ or of ‘most value’.^83^ In these scenarios, trade-offs between goods are permitted, where something can be sacrificed if it increases the aggregate, justified according to the logic of maximisation.

In contrast, claims of incommensurability involve the attachment of intrinsic value to certain goods—where value is derived purely from traits internal to the particular good. The value of our partner or our child is inherent to their very being, their particular traits, and so on: we do not need to appeal to other sources to justify why we value them. Indeed, an appeal to some external justification to explain their value would appear odd. If one stated, ‘I care for my child because of the status I gain from my friends’ or ‘I love my partner because of the tax deductions’, it would be a valid question to ask whether one truly valued one’s child or partner. Not only would this type of reasoning be seen as potentially abhorrent, but it would be criticised as an improper expression of *what it means* to respect, love, esteem, and care for something (ie intrinsic to the evaluative attitude itself) as well as what it means to respect, love, esteem, or care for *something* that ought to be valued for its own sake (ie prescribed by the very nature of the valued good). Intrinsic values are therefore guided by attitudes which have a *distributive* form, focusing on the assignment of status rather than weight or aggregative value to goods. This distributive principle resists making trade-offs in the name of maximisation and, instead, directs us ‘to distribute our different kinds of concern to each of the intrinsic goods in our lives. Thus, choices concerning those goods or their continued existence do not generally require that we rank their values on a common scale or choose the more valuable good; they require that we give each good its due’.^84^ Needless to say, what and how we give each good its due operates within a sphere of intersubjectivity in which its practical dynamics and consequences will be liable to difference, disagreement, or indeed consensus, depending on when the ranking is performed by a person on her own behalf or by another (say, a judge) for P, and how that ranking is generated and justified. Yet beneath these potentially conflicting views will often be a common distributive attitude in how the goods at stake are valued.

## V. RECONFIGURING JUDICIAL REASONING ABOUT BEST INTERESTS

Importantly, the deliberative nuances of judicial decision-making about best interests affirm the normative significance of the distributive principle outlined above. Put negatively, inappropriately adopting an aggregative attitude towards intrinsic goods equates with a failure to give each good that which it is due. This is where the analogy or metaphor of the balance sheet, and indeed the very terminology of the ‘best’ in best interests, goes awry, particularly in the temptation to interpret claims of status by reference to weight, believing that ‘in an ideal world, a best interests decision would be able to enhance all these aspects’,^85^ or that, as framed in a *dictum* quoted above, as a matter of principle ‘the best interests test ought, logically, to give only one answer’.^86^ The reality is that the framework of the law as it stands means that the judge is *required* to make a decision on behalf of the person who is deemed to lack capacity.^87^ But though a decision *has* to be made, irreconcilable tensions between equally strong arguments can mean not all dimensions of best interests pull in the same direction, even in cases where some broad overlapping consensus might be achieved.^88^ To return to Sir Mark Hedley’s example, it may well be the case the judge places the older adult in a care home, prioritising physical protection over his emotional comfort. Others might take the view that this is not the best decision that could have been reached, but its justification necessarily demands the judge to adopt a distributive attitude in light of the *nature* of the goods being evaluated and more importantly, the *obligation* that must be discharged to the individual as a bearer of rights.^89^ The judge—if she is not to invite legal appeal or public criticism—has to express her attunement to the gravity of the choice in the individual’s life, as well as the significance of her obligation to that individual. As Pildes writes, ‘[e]fforts to avoid the difficulties posed by treating values as sometimes incommensurable fail to acknowledge the way social relations and individual experiences depend upon appreciating values in certain ways’.^90^

How then should a judge reason practically such that she approaches the relevant goods and values by displaying the correct distributive attitude? Here, a broader scope of practical reasoning in best interests decision-making is required, both in terms of its defined function and the specification and evaluation of the permissible reasons that are invoked to settle the matter at hand. On one hand, the appeal of balancing lies precisely in reducing the practical reasoning process to a fairly simple rational calculus of various options. When one adopts this position, so long as all the inputs are accurately accounted for and the calculations are consistent, a single ‘right’ answer to the problem is possible. However, there might not be one right answer for the parties or even for P herself. Should we be more concerned with a person’s physical health rather than the intimate relationship that harms her? Do the person’s religious views trump preserving her life? Physical, mental, emotional, spiritual, and relational dimensions all jostle with one another in best interests decision-making, and they do so precisely because the individual in question is leading a life that is characterised fundamentally by multiple, divergent intrinsic values.

It might be thought that this leaves judges in an invidious position, not least as they cannot simply decline to make a decision. And in so far as critical reflection is consequently invited, we might welcome, for example, the candour expressed by Jackson J in *A Local Authority v E*. Following a best interests assessment that included an explicit representation of and engagement with a balance sheet, but prior to presenting his conclusion, the judge stated: ‘The balancing exercise is not mechanistic but intuitive and there are weighty factors on each side of the scales.’^91^ The foundations to such a statement are likely explained by our reasoning above, and the frank admission of the place of intuitions is admirable in its honesty.^92^ The question that follows, however, is whether recourse to a judge’s intuition is inevitable and desirable. Even if someone were to contest the claim of an inevitable resort to intuition, the above reflections suggest that the adjudicative functions prescribed by the MCA place demands on judges (as they do with other best interests decision-makers) to draw from and apply values that are contingent, unsettled, and inherently subject to potential disagreement. In regard to the practical reasoning conducted by judges in the process of arriving at a *legal* determination, this is not a matter that even in principle may be resolved by exclusive reference to the law. If there is such a thing as a satisfactory best interests determination, it is by its nature not something that may be achieved, even in principle, without methods of reasoning that expand beyond ‘the legal’. ‘Neat’ resolution, in the senses that are espoused even within idealised accounts of the exercise of judicial wisdom, are not theoretically possible. And this in turn serves as a call for the development of methods of understanding and reasoning within judicial practice that are, in formalist senses, alien to the law.

The reality of ‘the hard choice’ therefore rightly challenges the narrow procedural function of practical reasoning that is implied in the balance sheet, which presumes that a single, right answer can be generated by following a formal deliberative mechanism. Indeed, as judges are aware, in discharging their task of best interests decision-making, a judge must engage in difficult but crucial evaluative work prior to making any rational calculus. This is because the complex, multi-faceted constitutive elements of best interest judgments almost always involve appeal to intrinsic values. To make a decision in such cases, judicial reasoning must draw on language that goes beyond the fulfilment of contingent ends or desires, an approach that invokes a vocabulary of qualitative evaluations, such as dignity, worth, quality of life, or contrastive distinctions of higher and lower, good or bad, noble or ordinary, and so on.^93^ Such evaluative language is often bracketed due to fears that admitting its decisive role would imperil the status of judges as impartial, objective observers, effectively allowing them to make ‘subjective’ or ad hoc, poorly justified decisions which are then illegitimately imposed onto the lives of persons with disabilities. This worry tracks the widespread liberal intuition that such individuals in public roles have an obligation to appeal only to reasons that are putatively value-neutral.^94^ Yet, this objection is mistaken on two grounds. First, it is wrong to immediately characterise evaluative judgments and their grounding as ‘subjective’. Certain substantive goods and values in mental capacity law (eg autonomy, right to life, health, and well-being) frame deliberations in judgments, and their priority is warranted through broad societal and intersubjective consensus. Secondly, there is the presumption that individuals can voluntarily shed themselves of their value frameworks. Not only does this fail to reflect the phenomenology of best interests deliberation as described by judges such as Sir Mark Hedley, but it removes the crucial material which functions as the basis of one’s evaluation. Ultimately, drawing on substantive value sources and our evaluations of worth is needed in order to decide between incommensurable goods. These evaluations do not exist in the abstract but come alive precisely through the ethical intuitions and conflicting values that are activated in such difficult cases.

This is not to say that any or all evaluations ought to enjoy automatic warrant or avoid critical scrutiny, and nor is it to suggest that merely speaking the language of values will be sufficient. Indeed, as Taylor shows, qualitative evaluation embeds the necessary condition of *articulacy*. When faced with incommensurable options, invoking the vocabulary of worth helps advance articulations as to why one proposition or reason is found to be more elevated or superior to another.^95^ Articulations seek to convey what might be implicit, confused, or inchoate. They contain their own normative telos, in as much there is an aspiration for better, clearer, more comprehensible descriptions of the predicament at hand and the qualitative evaluation of the goods at stake.^96^

Aspirations for greater articulacy are poorly served by the mechanistic framework of the balance sheet. A stark example is provided in *Royal Bournemouth and Christchurch Hospitals NHS Foundation Trust v TG*, a case which revolved around the question of whether an endotracheal tube should be removed from a woman who was in a vegetative state, or whether it was in her best interests that it should be continued. Perhaps implicitly observing its limited function, Cohen J himself stated that ‘I have had to do’ the balance sheet exercise.^97^ He listed the various benefits and disbenefits accordingly:

What on the one hand is the benefit of removal of the tube? First, it would be the end of the process which brings, or is likely to bring no significant benefit to TG. Secondly, it removes the possibility of indignity and/or pain. I do not think there is more to be put in that column.

On the other side there is the continuation of life; there is the recognition of her wishes for herself and for her family; thirdly, it enables her life to progress and be ended in accordance with the will of God; fourthly, it permits the possibility, faint though it may be, of some improvement in her state and fifthly, although this may be repetitious, it provides the ability for her to play a part in her family as she and they would wish, even though she would be unaware of it.^98^

Cohen J concluded:

I have come to the clear decision that it is in the patient's best interests that intubation should continue. I recognise that this places a huge burden on the treating team. It is against their advice and their wishes … but I remind myself constantly, this is her life and her wishes as I have found them to be and nobody else's.^99^

Notably, the balance sheet does little to illuminate the determinative reasons behind the judge’s decision, nor can it function to advance the condition of articulacy in the sense that has been discussed. The various listed reasons on each side appear incommensurable, but which reason is to be elevated over others is unclear. On its own, the balance sheet merely sets out the incommensurable options rather than assisting to clarify a pathway of deciding between them. It is only *outside* the balance sheet that we learn of which reason has priority—namely TG’s life and wishes. Elsewhere in the judgment, Cohen J further articulates the importance of the continuance of life according to the wishes and feelings of TG and her family. Drawing on the statement of her husband and children, he outlined two strands:

[F]irst, that if her presence was a comfort to others (as I find it to be) she would want to be there whatever the cost to her. Family was central to her and she would want to remain a part of the family no matter what form it would take for as long as possible. Secondly, she had the utmost respect for life because of its intrinsic value and that it was for no-one other than the Lord to take away. It is for Him alone to end and she would never accept anyone else facilitating death. I also take into account the statement of her friend M who had a discussion with her about Dignitas in the context of a programme on television and she recalls TG saying, “Why do people want to go?” before adding something like “They're not God and they don't know what will happen in the future.” It is absolutely clear from everything that I have read that her Catholic faith and her belief in God were and are a crucial part of her life.^100^

One might argue that Cohen J’s decision merely reflects the requirements in section 4 of the MCA, which stipulate that determinations of best interests must take into consideration the values, wishes, beliefs, and feelings of P. At one level, this is true. At another level, however, the elevation of TG’s wishes and feelings works towards advancing judicial articulacy about a normative framework which recognises and elevates the status of P’s perspective—and not simply P’s expressed subjective preferences (whatever these may be) but more specifically, P’s own contrastive, higher-order evaluations that confer depth and meaning to his or her life overall. For instance, Cohen J made a point of endorsing the Official Solicitor’s position statement that TG would see her treatment in intensive care as aligned with her notions of the dignity of life and divine providence.^101^ Here, judicial qualitative evaluations are attuned to P’s own vocabulary of worth. Indeed, the condition of articulacy carries with it an associated requirement to track back and report precisely how and why a particular value or good emerges in the context of P, in the circumstances of the case, as being of evaluative significance worthy of articulation. Ways to focus this dialectical process of deliberation might be to focus on questions of *status* (‘what is it about the person and her life that can be identified as being of value?’); *magnetic importance* (‘what values concerning the person and her life stand out as being of particular significance?’), and *the narrowing down of reasons* (‘given the person’s circumstances how do the values of particular significance provide reasons for acting in a particular way on her behalf?’). In incorporating this dialectical process, the approach adopted by Cohen J thus attends to any residual concern that reasoning outside the framework of a balance sheet risks undermining the procedural legitimacy of the judgment.^102^ Best interests judgments can be challenged, appealed, and reviewed accordingly through further deliberative scrutiny in ways that accord with well-established legal processes.

Not all best interests cases will prioritise the values of P, and this is especially evident in some controversial circumstances where to do so would lead to her death.^103^ Attempts at greater articulacy are particularly important in interpreting the normative direction of a legislative framework which better gestures towards respect for P’s values, wishes, beliefs, and feelings, as situated within the context of other values. The particularistic and circumstantial nature of best interests, combined with the incommensurable values and goods at stake, suggest that the articulations of judges have a crucial part in making explicit and influencing the normative direction of best interests judgments made under the provisions of section 4 of the MCA. All articulations are not equal: some might be misguided, wrong, distortive, while others may be more comprehensible and foster greater clarity. But such debate can only be had when the misleading presuppositions of the balance sheet are dislodged in favour of an account of practical reasoning which recognises the necessity of engaging with qualitative evaluations as the basis of sound judicial decision-making about a person’s best interests.

## VI. CONCLUSION

The balance sheet has an intuitive attraction to many decision makers under the MCA, and not just judges themselves. As a template it has been adopted as a widespread practical tool for legal, and social and health care practitioners as an essential part of their section 4 best interests checklist for the (vast majority) of situations that are addressed outside the courts. It seems to allow the reader of a judgment,^104^ or best interests assessment, to see *what* the best interests decision maker has considered to be relevant, and *that* they have considered relevant factors. But, based on the analysis of this article, we suggest that little survives of the balance sheet as a deliberative mechanism for deciding best interests at the level of theory and practice. Even defined nominally as an ‘aide memoire’ of the factors to be considered, or as a placeholder for the deliberative act of ‘balancing’ and ‘weighing’, it is only one tool amongst many in making practical judgments around competing goods and values at stake, and cannot displace the more substantive deliberative processes at play. Indeed, and as many of the judges themselves recognise in different ways, the balance sheet does not in fact assist them in reaching the decisions that they are required to reach.

This critique of the balance sheet is important for a number of reasons: first, it means analysis of mental capacity case law can be more precisely focused on the implicit phenomenology of values-based engagement in judicial deliberation about best interests.^105^ Often the presumption is that the act of balancing is sufficient explanation of the reasoning behind a best interests decision—yet as shown above, it obscures the actual process of considering, evaluating, attaching significance to certain factors over others. Secondly, our critique challenges the allure of mechanistic reasoning and reveals how such a model of deliberation chafes against the distributive attitude that is demanded by such difficult cases involving competing intrinsic values in mental capacity law. Whether a person’s emotional or physical well-being takes priority, whether her autonomy trumps her safety—these fraught issues resist the language of trade-offs and demand an orientation of valuing in ways that recognise their nature as intrinsic goods. Finally, and importantly, the grounding and task of best interests decision-making is reframed accordingly: setting aside the illusion of the balance sheet, with its aggregative presumptions of commensurability, of quantitative comparison, makes room for an alternative grounding which revolves around the qualitative evaluations about conflicting, often incommensurable values; and a grounding that focuses on how these evaluations might be identified and drawn into consideration. Rather than lead to a domain of ‘ad hocery’ and subjective imposition, we would suggest that making explicit the significance of evaluative judgments could help make best interests assessors more adept at recognising and becoming more transparent about the depth of goods and values at stake, as well as the different reasons that frame one’s valuations.

This reframing is both urgent and timely: at the time of writing the MCA Code of Practice is currently being revised and reference to the balance sheet ought to be critically reconsidered in light of our analysis, especially considering how the Code continues to inform the adoption of the balance sheet template in practical guidelines for non-judicial best interests assessors. Though the statutory framing of the MCA requires judges to make concrete best interests decisions in court proceedings, as well as assessors to make decisions outside the courtroom, certainty of the ‘right’ answer nonetheless often can and will remain elusive. Such uncertainty gives way to further challenges in cases where, even in principle, there is no single ‘right’ answer; no unique and preclusively ‘best’ determination. But such uncertainty is to be welcomed, as the normative focus then becomes one of working towards greater articulacy about a best interests framework that recognises and accords status towards P’s own qualitative evaluations, while simultaneously appreciating that such decisions will inevitably be situated within a broader complex nexus of values and value sources.

## Footnotes

1 The device is also used in family law cases, both before the Family Division of the High Court and in the Family Court. However, our focus in this article relates to the balance sheet as a deliberative tool in Court of Protection cases (and mental capacity law cases concerning adults decided under the inherent jurisdiction of the High Court prior to the coming into force of the Mental Capacity Act 2005). For an early critical analysis comparing best interests under the Children Act 1989 and the (then) new MCA, see S Choudhry, ‘Best Interests in the MCA 2005: What can Healthcare Law Learn from Family Law?’ (2008) 16 Health Care Analysis 240. In relation to child and family law, see also A Daly, ‘No Weight for “Due Weight”? A Children’s Autonomy Principle in Best Interest Proceedings’ (2018) 26 Intl J Children’s Rights 61.

2
*Re A (Male Sterilisation)* [2000] 1 FLR 549 560 F-H.

3 Crucially, the adoption of the balance sheet has not been accepted across the board amongst the judiciary. Former Senior Judge Denzil Lush was particularly resistant to this approach.

4 cf s 1(3) of the Children Act 1989 which requires the court to have regard to seven statutory criteria (referred to by practitioners as the ‘welfare checklist’) when making certain decisions in the best interests of a child.

5 Setting out a series of steps that must be taken and factors to be considered by a person determining what is in another’s best interests for purposes of the Act.

6
*Re F (A Child) (International Relocation Cases)* [2015] EWCA Civ 882 at [52].

7
*Re N* [2015] EWCOP 76 at [52].

8
*NHS Foundation Trust v QZ* [2017] EWCOP 11 at [25].

9
*Re GM; FP v GM and A Health Board* [2011] EWHC 2778 (COP) at [24].

10 By way of examples only, see Suffolk Safeguarding Adults Board, which refers to Thorpe LJ in *Re A* and Greater Manchester Mental Health NHS Trust, *Mental Capacity Act and Deprivation of Liberty Safeguards Policy*, where it states, ‘In taking best interests decisions in court, Judges have often used the ‘balance sheet’ principle, drawing up a list of the emotional, medical, social and welfare benefits and disadvantages (including the likelihood of each benefit or disadvantage occurring) of the proposed alternatives’ (s 7). The balance sheet approach is likewise used as part of the training of the MCA for social workers, see SE Laird, *Practical Social Work Law: Analysing Court Cases and Inquiries* (Routledge 2010) 300. More explicitly, in H Carr and D Goosey, *Law for Social Workers* (15th edn., OUP 2019) 611, the balance sheet is discussed as part of the social worker’s toolkit and ‘use of [the balance sheet document] is recommended by the President of the Family Division, the Association of Directors of Children’s Services, Cafcass, HM Courts and Tribunals Service, the Department of Education, the Ministry of Justice, and the Chair of the Family Justice Board, in compliance with the revised Public Law Outline (PLO) 2014’. A quick search reveals numerous other practice guidelines where the balance sheet is endorsed, including the Local Government Association and ADASS, *Putting the Mental Capacity Act principles at the heart of adult social care commissioning: A guide for compliance*; Wales Health Board, Lincolnshire County Council; West Sussex County Council; Manchester Safeguarding Adults Board, *Mental Capacity Act Policy and Procedures* where, in Appendix 4, the balance sheet is ‘[r]ecommended for all professionals undertaking sophisticated decisions, particularly where the benefits are finely balance or in instances where there may be disagreement’.

11 For further critical discussion of the best interests standard, both in relation to mental capacity law and as it relates to children, see M Dunn and others, ‘Constructing and Reconstructing ‘best interests’: An Interpretative Examination of Substitute Decision-making under the Mental Capacity Act 2005’ (2007) 29 J Social Welfare & Family L117; J Coggon, ‘Mental Capacity Law, Autonomy, and Best Interests: An Argument for Conceptual and Practical Clarity in the Court of Protection’ (2016) 24 MLR 396; M Donnelly, ‘Best Interests, Patient Participation and the Mental Capacity Act 2005’ (2009) 17 MLR 1; M Donnelly, ‘Best Interests in the Mental Capacity Act: Time to Say Goodbye?’ 24 MLR 318; LM Kopelman, ‘The Best Interests Standard for Incompetent or Incapacitated Persons of all Ages’ (2007) 35 J L, Med & Ethics 187; D Degrazia, ‘Value Theory and the Best Interests Standard’ (1995) 9 Bioethics 50.

12 n 3 above.

13
*Re F (Mental Patient: Sterilisation)* [1990] AC 1.

14 n 3 above at [559].

15 Law Commission, *Mental Incapacity: Law Commission Report* 231 (Law Commission 1995).

16 Lord Chancellor’s Department, *Who Decides? Making Decisions on Behalf of Mentally Incapacitated Adults* (Lord Chancellors’s Department 1997).

17 n 3 above at [560].

18 The review was carried out through searches on Westlaw, as the single most comprehensive database of judgments. Some cases had neutral citations suggesting that they were decided in the Family Division or Chancery Division (an example being *Re P* [2009] EWHC 163 (Ch)) but were, in fact, Court of Protection cases. To be clear, this search was not carried out for the purposes of conducting a systematic review of the case law but to provide a comprehensive and useful overview of how the balance sheet has evolved in judicial decision-making. Our methodology reflects this purpose and the analysis of our article does not hinge on a systematic review of cases.

19
*A NHS Trust v K* [2012] EWHC 2922 (COP); (2013) 129 BMLR 61 at [45] per Holman J.

20
*Re S (Adult's lack of capacity: carer and residence)* [2003] 2 FLR 1235 at [14], where Wall J held that the approach of Thorpe LJ was ‘authoritative’ guidance for the court should go about deciding which outcome would best serve the welfare of the ‘mentally disabled person’.

21 See, eg *Westminster City Council v Sykes* [2014] EWCOP B9.

22
*Re JDS* [2012] EWHC 302 (COP); [2012] WTLR 475 at [23]. The ‘factor of magnetic importance’ was a term coined by Thorpe LJ in *Crossley v Crossley* [2007] EWCA Civ 1491, [2008] 1 FLR 1467.

23 See further the discussion in A Ruck Keene and others, ‘Taking Capacity Seriously? Ten Years of Mental Capacity Disputes before England’s Court of Protection’ (2019) 62 Int J Law Psychiatry 56.

24 See *Re G (TJ)* [2010] EWHC 3005 (Fam) at [37] per Morgan J.

25 See eg *Re CW* [2010] [2010] EWHC 3448 (Fam), the first reported treatment withdrawal case after the MCA 2005 came into force, relying upon *Airedale NHS Trust v Bland* [1993] AC 789. Lady Hale in *Aintree University Hospitals NHS Trust v James* [2013] UKSC 67; [2014] AC 591 noted at [37] that ‘[t]here are cases, such as Bland's case [1993] AC 789, where there is no balancing exercise to be conducted’.

26 See, eg *D v JC* [2012] 3 WLUK 800; [2012] WTLR 1211.

27 n 7 above at [52].

28
*Cambridge University Hospitals NHS Foundation Trust v BF* [2016] EWCOP 26; [2016] COPLR 411.

29
*Briggs v Briggs* [2016] EWCOP 53; [2017] 4 WLR 37, noting the need for a ‘holistic’ evaluation at [123].

30 n 9 above at [25].

31 ibid at [28].

32 The Code of Practice is under review at the time of writing.

33 n 11 above.

34 There are likely other comparable areas of judicial practice that we could draw from to illuminate the analysis in this article, but for the reasons presented in this section we find that debates on, and judicial reasoning concerning, proportionality are particularly instructive and of particular relevance.

35 P Craig, ‘Limits of Law: Reflections from Private and Public Law,’ in NW Barber, Richard Ekins and Paul Yowell (eds), *Lord Sumption and the Limits of the Law* (Hart 2016).

36 Human Rights Act 1998, s 6(3)(a).

37 ibid s 3.

38 M Hedley, *The Modern Judge: Power, Responsibility, and Society’s Expectations* (LexisNexis 2016) 53. See also *K v LBX* [2012] EWCA Civ 79.

39
*R (Burke) v General Medical Council* [2004] EWHC 1879 (Admin); *R (Burke) v General Medical Council* [2005] EWCA Civ 1003.

40
*R (Pretty) v DPP* [2001] UKHL 61; *R (Purdy) v DPP* [2009] UKHL 45; *R (Nicklinson) v Ministry of Justice* [2014] UKSC 38; *R (Conway) v Secretary of State for Justice* [2017] EWCA 2447.

41
*R (N) v Secretary of State for Health* [2009] EWCA Civ 795.

42
*R (Shelley) v Secretary of State for the Home Department* [2005] EWCA Civ 1810.

43 See eg J Rivers, ‘Proportionality and Variable Intensity of Review’ (2006) 65 Cambridge Law Journal 174; K Syrett, *The Foundations of Public Law: Principles and Problems of Power in the British Constitution* (2^nd^ edn, Palgrave: 2014) ch 5, especially 233–38; J Sumption, *Trials of the State: Law and the Decline of Politics* (Profile Books 2019).

44 [2001] UKHL 26.

45 ibid at [27].

46 ibid.

47
*R (Begum) v Denbigh High School Governors* [2006] UKHL 15.

48 ibid at [30].

49 R Alexy, ‘On Balancing and Subsumption’ (2003) 16 Ratio Juris 433.

50 M Jestaedt, ‘The Doctrine of Balancing – its Strengths and Weaknesses’ in M Klatt (ed), *Institutionalized Reason: The Jurisprudence of Robert* Alexy (OUP 2012) 157.

51 See FJ Urbina, ‘Proportionality in Medical Law’ in AM Phillips, TC de Campos and J Herring (eds), *Philosophical Foundations of Medical Law* (OUP 2019) 172; S Tsakyrakis, ‘Proportionality: An Assault on Human Rights?’ (2009) 7 International Journal of Constitutional Law 468, 474.

52 ibid 158.

53 PW Kahn, ‘The Court, The Community and the Judicial Balance: The Jurisprudence of Justice Powell’ (1987) 97 Yale LJ 1.

54 n 51 above; FJ Urbina, ‘Is It Really Easy: A Critique of Proportionality and Balancing as Reasoning’ (2014) 27 Can JL & Jur 167; FJ Urbina, ‘Incommensurability and Balancing’ (2015) 35 OJLS 575; VA da Silva, ‘Comparing the Incommensurable: Constitutional Principles Balancing and Rational Decision’ (2011) 31 OJLS 273.

55 Urbina (n 52); J Habermas, *Between Facts and Norms: Contributions to a Discourse Theory of Law and Democracy* (trans W Rehg, Policy 1996).

56 Tsakyrakis (n 51) 482.

57 See C Kong, ‘Beyond the Balancing Scales: The Importance of Prejudice and Dialogue in *A Local Authority v E & Ors*’ (2014) 26 Child & Fam LQ 216.

58
*R (N) v Secretary of State for Health* [2009] EWCA Civ 795.

59 ibid at [49], *per* Lord Clarke of Stone-cum-Ebony MR and Moses LJ. *Per* Keene LJ.

60 ibid at [72]. It should be noted that as well as the challenge to the Trust’s policy, referred to here, the judges held that for the same reasons the non-provision of an ongoing exemption in the regulations would have been proportionate for the same reasons.

61 ibid at [100].

62 ibid at [108].

63 J Waldron, ‘Fake Incommensurability: A Response to Professor Schauer’ (1994) 45 Hastings LJ 813. In challenging the presumption of commensurability, however, one need not be committed to a strong thesis of incommensurability, which adds the additional and more questionable claim of incomparability: that is, since no single metric applies to competing values, this means that they are likewise incomparable; or that attempts to compare them are destined to be irrational, arbitrary, or are experienced as tragic loss. Few subscribe to strong commensurability in this sense. We as individuals, let alone judges, constantly compare values and choices we technically cannot quantify. Sometimes tragic loss does accompany such choices, but not all the time. It is still the case that we do bring values into relation with each other, recommending weak incommensurability views like Waldron’s which assert that conflicting values or principles can still be subject to rational comparison and choice, even lexical ordering.

64 Indeed, some first-hand descriptions of judging seem to refer to balancing in this sense. See EW Thomas, *The Judicial Process: Realism, Pragmatism, Practical Reasoning and Principles* (CUP 2005).

65 K Möller, ‘Proportionality: Challenging the Critics’ (2012) 10 Intl J Constit L 709.

66 Urbina, ‘Is It Really Easy’ (n 54) 192.

67
*A and B v Rotherham Metropolitan Borough Council* [2014] EWFC 47.

68 ibid at [85].

69 Urbina, ‘Is It Really Easy’ (n 54) 181.

70 I Kennedy, ‘Patients, Doctors and Human Rights’ in R Blackburn and J Taylor (eds), *Human Rights for the 1990s* (Mansell 1991) 90.

71 ibid.

72
*Re S (Adult Patient: Sterilisation)* [2000] 3 WLR 1288, at 27.

73 J Raz, ‘Incorporation by Law’ (2004) 10 Legal Theory 1.

74 CR Sunstein, ‘On the Expressive Function of Law’ (1996) 144 UPA L Rev 2021.

75 n 39 above, 48.

76 Sir Mark Hedley does not mention P’s values, but we would argue that these are automatically activated in light of s 4 of the MCA.

77 n 39 above, 50–51.

78 ibid 54, 53.

79 ibid 65. See also CR Sunstein, ‘Incommensurability and Valuation in the Law’ (1994) 92 Mich L Rev 779, who also notes how welfare, utility, costs and benefits analyses seem to invite the assumption of measurement and exclude other, more diverse qualitative forms of evaluating goods in the law.

80 n 39 above, 54.

81 ibid.

82 Part of the difficulty in being able to identify the range of values (and value sources) does not just concern the lure of measurement in contemporary political and social discourse, but also the overarching philosophical difficulty in articulating, and doing conceptual justice to, the form and practical application of non-consequentialist values—a problem that is pervasive (not just in day-to-day practical moral reasoning, but in academic fields relating to health care and in bioethics itself), leading to the poverty of normative scholarship in these fields.

83 E Anderson, ‘Practical Reason and Incommensurable Goods’ in R Chang (ed), *Incommensurability, Incomparability, and Practical Reason* (Harvard University Press 1997) 103.

84 ibid 104.

85 n 39 above, 64.

86
*Re S (Adult Patient: Sterilisation)* (n 72) above, *per* Butler-Sloss P, at 27.

87 See *Secretary of State for Justice v KC and C Partnership NHS Foundation Trust (Mental health: All)* [2015] UKUT 376 (AAC) at para 37.

88 n 39 above, 64–65.

89 n 83 above, 106; also Sunstein (n 79).

90 RH Pildes, ‘Conceptions of Value in Legal Thought’ (1992) 90 Mich L Rev 1520, 1549.

91
*A Local Authority v E* [2012] 1639 (COP); [2012] HRLR 29, at [129].

92 Though Jackson J nonetheless uses the imagery of the scales in deciding in favour of the presumption of life, it is revealing how powerful the balancing analogy remains and how it prevents the explicit articulation of the intuitive judgments carrying the burden of his decision.

93 C Taylor, ‘What is Human Agency?’ in *Human Agency and Language* (CUP 1985) and C Taylor, ‘Explanation and Practical Reason’ in *Philosophical Arguments* (Harvard University Press 1995).

94 J Rawls, *A Theory of Justice* (Harvard University Press 2009 [1971]).

95 n 94 above. Others (n 74 above) highlight the ‘expressive’ function of the law in this sense. We follow Taylor in using the term ‘articulation’ rather than ‘expression’ due to the latter’s connotations of moral subjectivism in the history of philosophy.

96 Rawls (n 94).

97
*Royal Bournemouth and Christchurch Hospitals NHS Foundation Trust v TG & Anor* [2019] EWCOP 21 at [28].

98 ibid at [28–29].

99 ibid at [30].

100 ibid at [24].

101 ibid at [25].

102 ibid at [24].

103 Compare two best interests cases decided by Peter Jackson J (as he was then), for example *A Local Authority v E & Ors* [2012] EWHC 1639 (COP) and *Wye Valley NHS Trust v B* [2015] EWCOP 60. This is why, for example, our critique of the balance sheet approach does not attempt to replace it with an appeal to other blanket mechanisms or meta-principles which take the subjectivity of P as the deliberative starting and end point—such as an exclusive focus on P’s subjective will and preferences (as suggested in particular readings of art 12 of the United Nations Convention on the Rights of Persons with Disabilities), or a substituted judgment test—on grounds that such approaches likewise seem unable to deal with the complexity and incommensurability of values. Nor do they encourage the conditions of articulacy we discuss here.

104 As to whom judgments are addressed, including the place or absence of P as a participant in and addressee of a judicial determination, see further J Harrington, L Series and A Ruck Keene, ‘Law and Rhetoric: Critical Possibilities’ (2019) 46 J L & Society 302; J Harrington, *Towards a Rhetoric of Medical Law* (Routledge 2016).

105 As indicated above, we have not sought to relate the critical insights of this article to child and family law, but there is likely application within the distinct approaches to best interests decision-making within that sphere as well.

## Notes

We are grateful to the Arts and Humanities Research Council for its support through the *Judging Values and Participation in Mental Capacity Law* project (AHRC Grant Reference: AH/R013055/1). Many thanks to our colleagues on the project, Penny Cooper, Rebecca Stickler, Matthew Watkins, and Victoria Butler-Cole QC, for their comments and suggestions in relation to this article. Thanks also to colleagues at the 2019 Society of Legal Scholars Annual Conference’s Medical Law stream and the Medical Ethics and Law Discussion Group in the Faculty of Law, University of Oxford, particularly Jonathan Herring and Charles Foster. Finally, our appreciation to two anonymous reviewers for their feedback. The views expressed in the article are those of the authors.

